# Isolation of Fucosyltransferase-Producing Bacteria from Marine Environments

**DOI:** 10.1264/jsme2.ME12058

**Published:** 2012-10-26

**Authors:** Hitomi Kajiwara, Munetoyo Toda, Toshiki Mine, Hiroshi Nakada, Takeshi Yamamoto

**Affiliations:** 1 Glycotechnology Business Unit, Japan Tobacco Inc., 700 Higashibara, Iwata, Shizuoka 438–0802, Japan; 2 Intellectual Property Center, Legal Division, Japan Tobacco Inc., 2–1, Toranomon 2-chome, Minato-ku, Tokyo, 105–8422, Japan; 3 Department of Biotechnology, Faculty of Engineering, Kyoto Sangyo University, Motoyama, Kamigamo, Kita-ku, Kyoto, Kyoto 603–8555, Japan

**Keywords:** fucosyltransferase, lectin staining, marine bacteria

## Abstract

Fucose-containing oligosaccharides on the cell surface of some pathogenic bacteria are thought to be important for host-microbe interactions and to play a major role in the pathogenicity of bacterial pathogens. Here, we screened marine bacteria for glycosyltransferases using two methods: a one-pot glycosyltransferase assay method and a lectin-staining method. Using this approach, we isolated marine bacteria with fucosyltransferase activity. There have been no previous reports of marine bacteria producing fucosyltransferase. This paper thus represents the first report of fucosyltransferase-producing marine bacteria.

Fucose is usually the terminal monosaccharide in carbohydrate moieties of various glycoconjugates. The fucosylated carbohydrate chains of glycoconjugates play important roles in many biological processes, including fertilization, neuronal development, immune responses, and cell adhesion (9, 13, 17). The transfer of fucose to carbohydrate chains is performed by fucosyltransferases (10). Fucosyltransferases catalyze the transfer of L-fucose from the common donor substrate guanosine-5′-diphospho-β-L-fucose (GDP-fucose) to their acceptor substrates to form fucosides (10). Because α-fucosylated enzyme reaction products are formed from the β-fucosylated sugar nucleotide donor substrate GDP-fucose, all known fucosyltransferases are classified as inverting glycosyltransferases (10). To date, many fucosyltransferases have been obtained from various sources (10, 16). They are classified into five families according to the glycosidic linkages they synthesize: α1,2-fucosyltransferases, α1,3-fucosyltransferases, α1,4-fucosyltransferases, α1,6-fucosyltransferases, and *O*-fucosyltransferases (10). On the other hand, all fucosyltransferases, including putative enzymes, are classified into nine families on the basis of sequence similarities in the CAZy (carbohydrate-active enzymes) database (4): glycosyltransferase family (GT) 2, α1,2-fucosyltransferase from *Dictyostelium discoideum*; family 10, α1,3-fucosyltransferase and α1,4-fucosyltransferase from *Homo sapiens*, *Helicobacter pylori*, and others; family 11, α1,2-fucosyltransferase from *H. sapiens*, *H. pylori*, *E. coli*, and others; family 23, α1,6-fucosyltransferase from *Bradyrhizobium japonicum*, *Drosophila pseudoobscura*, and others; family 37, α1,2-fucosyltransferase from *Arabidopsis thaliana*, *Populus tremula*, and others; family 56, α1,2-fucosyltransferase from bacteria such as *E. coli*; family 65 and 68, *O*-fucosyltransferase from *H. sapiens*, *Danio rerio*, *Zea mays*, and others; and family 74, α1,2-fucosyltransferase from *D. discoideum*.

With regard to bacterial fucosyltransferases, only a few enzymes have been cloned and characterized. For example, α1,2-fucosyltransferases from *E. coli* and *H. pylori* show unique acceptor substrate specificities (20, 25–27). Putative bacterial α1,3- and α1,4-fucosyltransferases have been identified in *E. coli* (5), *Vibrio cholera* (24), *Rickettsia conorii* (14), *Salmonella enterica serovar Typhi* (18), and *Yersinia pestis* (19), but only α1,3/4-fucosyltransferase from *H. pylori* has been well characterized (1, 3). Some soil bacteria, such as *B. japonicum* (23), *Azorhizobium caulinodans* (12), *Mesorhizobium loti* (15), and *Rhizobium loti* (6), produce α1,6-fucosyltransferase.

Many studies have revealed that fucosylated carbohydrate chains are expressed on the cell surfaces of fucosyltransferase-producing bacteria. For example, fucosylated carbohydrate chains, such as the Lewis X structure (Gal-β-1,4-[Fuc-α-1,3-]GlcNAc) and the Lewis Y structure (Fuc-α-1,2-Gal-β-1,4-[Fuc-α-1,3-]GlcNAc), are expressed on the cell surface of *H. pylori* (2, 22, 29).

Because the fucosylated oligosaccharides of glyco-conjugates play important roles in many biological processes, an abundant supply of fucosides is essential for a detailed investigation of their biological functions. To produce these fucosides enzymatically, many kinds of fucosyltransferases in large quantities are essential. Generally, bacterial enzymes are stable and show broad acceptor substrate specificity compared with their mammalian counterparts. For these reasons, we have been screening bacteria for glycosyltransferase activities, including fucosyltransferase activity and galactosyltransferase activity.

The bacterial isolates used in the screening were prepared from samples of seawater, sand, mud, small animals, and seaweed collected from the shore of various locations in Japan. Bacteria that grew on marine agar 2216 at 15, 25, 28 or 30°C were isolated from the samples. Then, these bacteria were inoculated in a 15-mL test tube containing 6 mL of marine broth 2216 (Becton–Dickinson, Franklin Lakes, NJ, USA) and cultivated at 15, 25, 28, or 30°C for 18 h on a rotary shaker (180 rpm). Bacteria were harvested from 2 to 4 mL of the culture broth by centrifugation, suspended in 200 μL of 20 mM bis-Tris buffer (pH 6.0) that contained 0.2% Triton X-100, lysed by sonication on ice, and used immediately for the one-pot glycosyltransferase assay. To assess the enzymatic activities of various glycosyltransferases simultaneously, a one-pot glycosyltransferase assay was performed by mixing various donor substrates of glycosyltransferases with a mixture of acceptor substrates. The mixture of donor substrates of glycosyltransferases comprised guanosine-5′-diphospho-fucose, GDP-Fuc (Calbiochem, San Diego, CA, USA), uridine 5′-diphospho-galactose, UDP-Gal (Calbiochem), and uridine 5′-diphospho-*N*-acetylgulcosamine, UDP-GlcNAc (Sigma, St. Louis, MO, USA). GDP-[U-^14^C]-Fuc, UDP-[U-^14^C]-Gal, and UDP-[U-^14^C]-GlcNAc were from Amersham Biosciences (Little Chalfont, UK). The mixture of acceptor substrates of glycosyltransferases comprised 4-nitrophenyl α-D-galactopyranoside (Gal-α-pNp), 4-nitrophenyl β-D-galactopyranoside (Gal-β-pNp), 4-nitrophenyl *N*-acetyl-α-D-galactosaminide (GalNAc-α-pNp), 4-nitrophenyl *N*-acetyl-β-D-galactosaminide (GalNAc-β-pNp), 4-nitrophenyl *N*-acetyl-α-D-glucosaminide (GlcNAc-α-pNp), 4-nitrophenyl *N*-acetyl-β-D-glucosaminide (GlcNAc-β-pNp), 4-nitrophenyl α-D-glucopyranoside (Glc-α-pNp), 4-nitrophenyl β-D-glucopyranoside (Glc-β-pNp), 4-nitrophenyl α-L-fucopyranoside (Fuc-α-pNp), 4-nitrophenyl β-L-fucopyranoside (Fuc-β-pNp), 4-nitrophenyl α-D-mannopyranoside (Man-α-pNp), and 4-nitrophenyl β-D-mannopyranoside (Man-β-pNp). All of the acceptor substrates were purchased from Sigma. The reaction mixture for the one-pot glycosyltransferase assay (50 μL) consisted of the following: an enzyme sample, a mixture of 0.5 mM acceptor substrates consisting of 4-nitrophenyl compounds, a mixture of 0.5 mM donor substrates consisting of sugar nucleotides that included 4,620 Bq UDP-[U-^14^C]-Gal, 4,620 Bq UDP-[U-^14^C]-GlcNAc, and 4,620 Bq GDP-[U-^14^C]-Fuc, 100 mM bis-Tris buffer (pH 6.0), 10 mM MnCl_2_, and 3 mM ATP. The reaction was carried out at 25°C for 16 to 18 h. After the reaction, 100 μL water was added to the reaction mixture, and the mixture was applied to a Sep-Pak Vac C18 cartridge 1 cc/50 mg 55–105 μm (Waters, Milford, MA, USA) that was conditioned with ethanol and equilibrated with water. The column was washed twice with 1 mL water, and the reaction product was eluted with 1 mL of 70% ethanol. One milliliter of scintillation cocktail was added to the eluate, and the radioactivity of the mixture was measured using a liquid scintillation counter.

More than 1,000 bacterial isolates were examined for glycosyltransferase activity, some of which tested positive in the first screening using the one-pot glycosyltransferase assay (Table 1). Table 1 showed the results of the assay with a mixture of acceptor substrates and donor substrates in the reaction mixture. With regard to DOT-118-2 and OKI-895, radioactivities of the eluates containing the mixture of acceptor substrates in the reaction mixture were over 2,000 cpm and approximately 1,100 cpm, respectively. On the other hand, radioactivities of the eluates without the mixture of acceptor substrates in the reaction mixture were about 100 cpm and about 300 cpm, respectively; however, with or without the mixture of acceptor substrates in the reaction mixture, almost the same radioactivities of the eluates were observed in other bacterial cases. Thus, we assumed DOT-118-2 and OKI-895 to be candidate glycosyltransferase producers among the tested bacteria.

Then, DOT-118-2 and OKI-895 were tested for their ability to transfer fucose, galactose, and *N-*acetylglucosamine to acceptor substrates, independently. DOT-118-2 and OKI-895 were isolated from samples obtained from the shore of the Okhotsk Sea, Hokkaido, Japan and the Eastern China Sea, Okinawa, Japan, respectively. As a result, we confirmed that DOT-118-2 exhibited fucosyltrans-ferase activity (Table 2). No galactosyl-transferase activity or *N*-acetylglucosaminyltransferase activity was observed in the lysate prepared from DOT-118-2. With regard to OKI-895, very weak fucosyltransferase activity was observed under the test conditions used in this study. No galactosyltransferase activity or *N*-acetylglucosaminyltransferase activity was observed in the lysate prepared from OKI-895.

As the next screening step, lectin staining was carried out as previously reported (8). After cultivation, the bacterial cells were collected by centrifugation (8,000×*g*, 15 min, 4°C) and resuspended in 25 mM Tris-HCl buffer (pH 7.5). These suspensions were spotted onto glass slides (MAS-coated glass slides [glass slides coated with a material that prevents cell detachment]; Matsunami Glass, Osaka, Japan), fixed with a 4% (w/v) paraformaldehyde solution at room temperature for 10 min, and blocked with a 5% (w/v) bovine serum albumin (BSA) phosphate-buffered saline (PBS) solution. After the glass slides were washed, biotin-labeled *Aleuria aurantia* lectin (AAL; Seikagaku Kogyo, Tokyo, Japan) or *Ulex europaeus* agglutinin-I (UEA-I; Seikagaku Kogyo) was added at room temperature for 2 h, and the cells were washed four times with PBS. Alexa 594-labeled streptavidin solution (Invitrogen, Carlsbad, CA, USA, 5 μg mL^−1^) was added, and the incubation continued at room temperature for 1 h. After five washes with PBS, the cells were mounted using ProLong Gold Antifade Reagent (Invitrogen), and observed by means of differential interference contrast (DIC) microscopy and fluorescence microscopy. Here, AAL strongly recognizes L-fucose residue, and UEA-I strongly recognizes fucose residue of Fuc-α-1,2-Gal-β-1,4-GlcNAc structures on glyco-conjugates (7, 11, 28), respectively.

As shown in Fig. 1, AAL bound well to DOT-118-2 but not to OKI-895. These results indicate that L-fucose residues were much expressed on the cell surface of DOT-118-2 than that of OKI-895. On the other hand, UEA-I did not bind to either DOT-118-2 or OKI-895. These results strongly indicate that Fuc-α-1,2-Gal-β-1,4-GlcNAc structures are not expressed on the cell surface of either DOT-118-2 or OKI-895. From these results, it was expected that fucosyltrans-ferase would be expressed much more in DOT-118-2 than OKI-895. Thus, we tried to identify DOT-118-2 prior to OKI-895. To identify DOT-118-2, 16S rRNA analysis of the bacterium was carried out. Genomic DNA was isolated from bacterial pellets using Genomic-tip 500/G (Qiagen, Chatsworth, CA, USA). The PCR reaction mixture for amplifying DNA encoding the 16S rRNA of the target bacterium consisted of genomic DNA (approximately 1 μg), 10 pmol of primers (27f and 1525r), 4 μL of each dNTP (2.5 mM), 2.5 units of PyroBest DNA Polymerase (0.5 μL), and 5 μL of 10× PyroBest buffer, in accordance with the manufacturer’s instructions (Takara Bio, Shiga, Japan). The reaction was hot-started at 96°C for 3 min; incubated at 96°C for 1 min, 55°C for 1 min, and 72°C for 2 min for 35 cycles; and further incubated at 72°C for 6 min in the Program Temp Control System (ASTEK, Fukuoka, Japan). The PCR product was cloned into a pCR4Blunt topo vector (Invitrogen, Carlsbad, CA, USA) using the protocol provided by the manufacturer. The pCR4Blunt topo vector containing the PCR product was introduced into *E. coli* TB1. The plasmid containing a 1.6 kb-length inserted fragment was then analyzed for DNA sequencing. DNA sequences were determined using the dideoxy chain termination method with an ABI PRISM fluorometric autocycle sequencer (Model 310 Genetic Analyzer; Applied Biosystems, Foster City, CA, USA). The sequence was determined on both strands. DNA sequences were analyzed by Genetyx (Genetyx Corporation, Tokyo, Japan), and a database search was performed using the BLAST program in GenBank. The primers used for PCR and sequencing were as follows: 27f (5′-AGAGTTTGATCCTG GCTCAG-3′); 1525r (5′-AAAGGAGGTGATCCAGCC-3′); 9F (5′-GAGTTTGATCCTGGCTCAG-3′); 339F (5′-CTCCT ACGGGAGGCAGCAG-3′); 785F (5′-GGATTAGATACCC TGGTAGTC-3′); 1099F (5′-GCAACGAGCGCAACCC-3′); 536R (5′-GTATTACCGCGGCTGCTG-3′); 802R (5′-TACC AGGGTATCTAATCC-3′); 1242R (5′-CCATTGTAGCACG TGT-3′); 1510R (5′-GGCTACCTTGTTACGA-3′); 1541R (5′-AAGGAGGTGATCCA-3′).

A partial DNA sequence (accession number: AB71119) of the 16S rRNA gene of DOT-118-2 showed the highest similarity, over 98%, to the DNA sequence of the 16S rRNA gene from *Polaribacter* sp. S-6 (accession number DQ978987). The result of phylogenetic tree analysis of the 16S rRNA gene is shown in Supplementary Figure 1. Further biochemical characterization of this bacterium is underway.

Recently, it has been reported that marine bacteria including Polaribacter produce exopolysaccharides, EPS, as a strategy for growth, adhering to solid surfaces, and to survive adverse conditions (21). It has been also reported that EPS are composed of monosaccharide such as D-arabinose, D-xylose, D-glucose, D-galactose, D-mannose, L-fucose, D-glucosamine, D-galactosamine, and so on; however, biosynthesis of EPS has not been understood comprehensively. The structure and composition of EPS produced by DOT-118-2 and OKI-895 are still unknown, and there is a possibility that the fucosyltransferase activities reported in this paper might be involved in the biosynthesis of EPS.

We screened marine bacteria for glycosyltransferase activities using two methods, the one-pot glycosyltransferase assay and lectin staining. As a result, we obtained two fucosyltransferase-producing bacteria from marine environments. Although enzymatic characterization of the fucosyltransferases produced by DOT-118-2 and OKI-895 has not been performed yet, there is a possibility that these enzymes would be good tools for producing fucosides enzymatically in the near future.

## Supplementary Material



## Figures and Tables

**Fig. 1:**
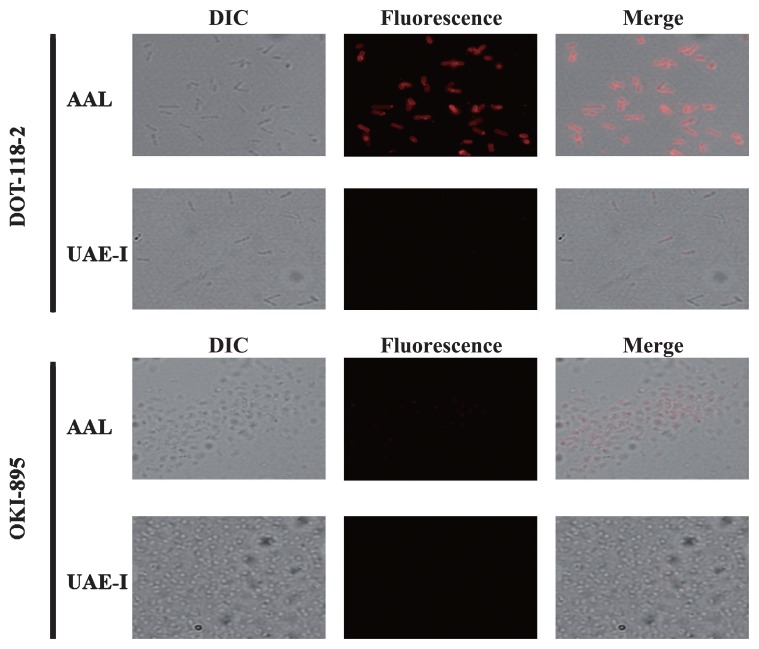
Detection of fucose residues on marine bacterial cells using biotin-labeled *Aleuria aurantia* lectin and *Ulex europaeus* agglutinin-I staining. DIC: Results of observations by differential interference contrast microscopy; Fluorescence: Results of *Aleuria aurantia* lectin and *Ulex europaeus* agglutinin-I staining observed by means of fluorescence microscopy; Merge: Merged DIC and fluorescence results.

**Table 1:** Results of the one-pot glycosyltransferase assay

Radioactivity (cpm)	No. of bacteria
<100	744
100–199	260
200–399	60
400–999	43
1,000–1,999	5
>2,000	1
total bacteria	1,113

The enzyme reaction was performed for more than 20 h at 25°C.

**Table 2:** Donor-substrate specificity of the glycosyltransferase in strains DOT-118-2 and OKI-895

Donor	Radioactivity (cpm)†	

	DOT-118-2	OKI-895
control (without acceptor)	32	30
UDP-GlcNAc	19	26
UDP-Gal	36	79
GDP-Fuc	3,456	278

† The enzyme reaction was performed for 18 h at 25°C.
