# Author’s Correction

**DOI:** 10.1264/jsme2.ME12058e

**Published:** 2013-09-21

***Short Communication***

**Isolation of Fucosyltransferase-Producing Bacteria from Marine Environments**

Hitomi Kajiwara^1,2^, Munetoyo Toda^3^, Toshiki Mine^1^, Hiroshi Nakada^2^, and Takeshi Yamamoto^1^*

^1^*Glycotechnology Business Unit, Japan Tobacco Inc., 700 Higashibara, Iwata, Shizuoka 438–0802, Japan;* ^2^*Intellectual Property Center, Legal Division, Japan Tobacco Inc., 2–1, Toranomon 2-chome, Minato-ku, Tokyo, 105–8422, Japan; and* ^3^*Department of Biotechnology, Faculty of Engineering, Kyoto Sangyo University, Motoyama, Kamigamo, Kita-ku, Kyoto, Kyoto 603–8555, Japan*

Volume 27, no. 4, Page 515–518, 2012

Page 517, ‘A partial DNA sequence (accession number: AB**711119**) of the 16S rRNA gene of DOT-118-2 showed the highest similality, over 98%, to the DNA sequence of the 16S rRNA gene from *Polaribacter* sp. S-6 (accession number DQ978987)’.

