# Supplementary material for: Isolation of Fucosyltransferase-Producing Bacteria from Marine Environments
Source: Microbes Environ. 2012 Oct 26;27(4):515–8. doi: 10.1264/jsme2.ME12058 (PMC4103564; doi:10.1264/jsme2.ME12058)
Supplement: Supplementary file 1 [file 27_515_s1.pdf]

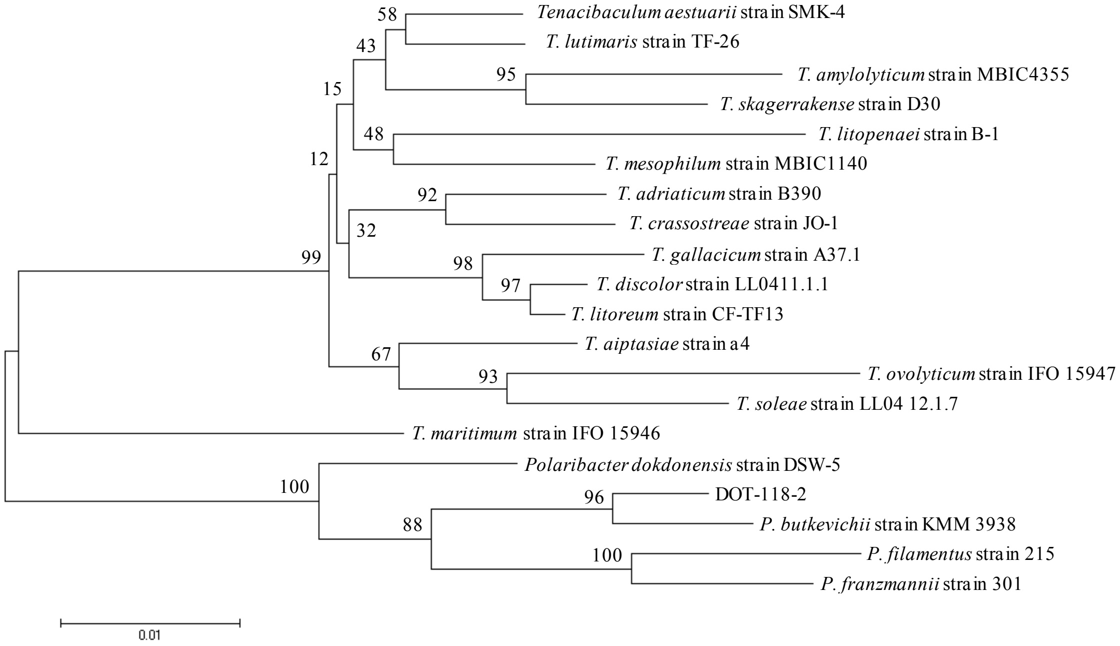

Fig. S1 Phylogenetic tree analysis of the 16S rRNA gene from strain DOT-118-2.

The evolutionary history was inferred using the Neighbor-Joining method [1]. The bootstrap consensus tree inferred from 1000 replicates [2] is taken to represent the evolutionary history of the taxa analyzed [2]. Branches corresponding to partitions reproduced in less than 50% bootstrap replicates are collapsed. The percentage of replicate trees in which the associated taxa clustered together in the bootstrap test (1000 replicates) are shown next to the branches [2]. The tree is drawn to scale. The analysis involved 20 nucleotide sequences. All positions containing gaps and missing data were eliminated. There were a total of 1366 positions in the final dataset. Evolutionary analyses were conducted in MEGA5 [3].

References

[1] Saitou, N. and Nei, M. 1987. The neighbor-joining method: A new method for reconstructing phylogenetic trees. Molecular Biology and Evolution 4:406-425.

[2] Felsenstein, J. 1985. Confidence limits on phylogenies: An approach using the bootstrap. Evolution 39:783-791.

[3] Tamura, K., Peterson, D., Peterson, N., Stecher G., Nei M., and Kumar S. 2011. MEGA5: Molecular Evolutionary Genetics Analysis using Maximum Likelihood, Evolutionary Distance, and Maximum Parsimony Methods. Molecular Biology and Evolution 10:2731-2739.
